# The influence of sex and diet on the characteristics of hibernation in Syrian hamsters

**DOI:** 10.1007/s00360-017-1072-y

**Published:** 2017-03-21

**Authors:** Marie Trefna, Maaike Goris, Cynthia M. C. Thissen, Vera A. Reitsema, Jojanneke J. Bruintjes, Edwin L. de Vrij, Hjalmar R. Bouma, Ate S. Boerema, Robert H. Henning

**Affiliations:** 1 Department of Clinical Pharmacy and Pharmacology, University Medical Center Groningen, University of Groningen, Hanzeplein 1, 9713 GZ Groningen, The Netherlands; 2 Departments of Chronobiology and Molecular Neurobiology, Groningen Institute for Evolutionary Life Sciences, University of Groningen, Nijenborgh 7, 9747 AG Groningen, The Netherlands; 3 Department of Internal Medicine, University Medical Center Groningen, University of Groningen, Hanzeplein 1, 9713 GZ Groningen, The Netherlands

**Keywords:** Hibernation, Mesocricetus auratus, Torpor, Sex differences, Linoleic acid, Polyunsaturated fat

## Abstract

Research on deep hibernators almost exclusively uses species captured from the wild or from local breeding. An exception is Syrian hamster (*Mesocricetus auratus)*, the only standard laboratory animal showing deep hibernation. In deep hibernators, several factors influence hibernation quality, including body mass, sex and diet. We examined hibernation quality in commercially obtained Syrian hamsters in relation to body mass, sex and a diet enriched in polyunsaturated fatty acids. Animals (M/F:30/30, 12 weeks of age) were obtained from Harlan (IN, USA) and individually housed at 21 °C and L:D 14:10 until 20 weeks of age, followed by L:D 8:16 until 27 weeks. Then conditions were changed to 5 °C and L:D 0:24 for 9 weeks to induce hibernation. Movement was continuously monitored with passive infrared detectors. Hamsters were randomized to control diet or a diet 3× enriched in linoleic acid from 16 weeks of age. Hamsters showed a high rate of premature death (*n* = 24, 40%), both in animals that did and did not initiate torpor, which was unrelated to body weight, sex and diet. Time to death (31.7 ± 3.1 days, *n* = 12) or time to first torpor bout (36.6 ± 1.6 days, *n* = 12) was similar in prematurely deceased hamsters. Timing of induction of hibernation and duration of torpor and arousal was unaffected by body weight, sex or diet. Thus, commercially obtained Syrian hamsters subjected to winter conditions showed poor survival, irrespective of body weight, sex and diet. These factors also did not affect hibernation parameters. Possibly, long-term commercial breeding from a confined genetic background has selected against the hibernation trait.

**Electronic supplementary material:**

The online version of this article (doi:10.1007/s00360-017-1072-y) contains supplementary material, which is available to authorized users.

## Introduction

Hibernation developed in endotherms to minimize energy expenditure of maintaining a constant body temperature to cope with challenging environmental conditions such as cold or drought and scarcity of supplies. Hibernation is a broadly conserved evolutionarily trait present in most mammalian lineages (Carey et al. 2003; Heldmaier et al. 2004; Melvin and Andrews 2009). Hibernation in rodents constitutes of two phases: torpor and arousal. Torpor is characterized by depressed metabolism to 1–5% of basal metabolic rate and a marked decrease in body temperature, heart rate and activity, which can last for hours, days, or even months (Storey 2010; van Breukelen and Martin 2002). Torpor is regularly alternated with short periods of complete restoration of metabolism resulting in normothermia, referred to as arousal. Interestingly, these torpor and arousal periods, featuring rapid cooling and rewarming, do not cause any apparent organ damage. In contrast, in non-hibernators such physiological extremes lead to the induction of cell death and subsequent organ damage (Zancanaro et al. 1999; Arendt et al. 2003; Talaei et al. 2011). Consequently, mechanisms deployed by hibernators attract interest from the medical field, because of their capacity to lower metabolism and alleviate organ damage by enduring oxidative stress (reviewed in: Bouma et al. 2012). Specifically, induction and maintenance of a torpor-like state in humans holds promise to lower morbidity and mortality in conditions such as trauma, cardiac arrest, organ transplantation and major surgery.

Deep hibernators, i.e., species that show multi-day torpor bouts, that are studied in the laboratory setting represent a large range of animals (Lyman et al. 1982; Geiser and Ruf 1995; Heldmaier et al. 2004) For this purpose, most species are captured from the wild and subsequently used in experimentation. A notable exception to this is the Syrian hamster (*Mesocricetus auratus*), the only deep hibernator belonging to the commercially available standard laboratory animal species, originally captured in the vicinity of Aleppo, North-West Syria in 1930 and originating from 3 to 4 littermates (Hoosier and McPherson 1987). Because of the latter, their genetic base is narrow. In addition, their prolonged commercial breeding, targeting maximal offspring in the minimum amount of time, may have selected against the hibernation trait. The majority of deep hibernators studied under laboratory conditions represent seasonal hibernators. Seasonal hibernators are usually fat storing, thus depending on the buildup of extensive fat reserves prior to hibernation, which is then orchestrated by an endogenous circannual rhythm (Jansky et al. 1984; Tamura et al. 2005; Florant and Healy 2012). In contrast, Syrian hamster is a facultative hibernator able to enter hibernation when conditions require so, increasing only moderately in weight prior to hibernation and hoarding food stocks to feed in between torpor bouts (Humphries et al. 2001). The facultative nature of deep hibernation in Syrian hamsters makes them suitable to study hibernation under laboratory conditions, as hibernation may be induced in any season by exposure to a short-day photoperiod and cold ambient temperatures (Oklejewicz et al. 2001). Several factors have been identified that influence the quality of hibernation, particularly torpor bout duration, which represents the crucial parameter determining total energy saving (Lyman et al. 1981). Torpor bout duration also relates to other life history traits including reproduction and longevity (Oxberry 1979; Lyman et al. 1981; Turbill et al. 2011). More specifically, among the factors suggested to play a role in the wild setting are body mass prior to hibernation, sex, age, availability of resources and dietary composition (Siutz et al. 2016; Chayama et al. 2016; Contreras et al. 2014; Terada and Ibuka 2000). Body mass affects hibernation characteristics, as both fat-storing and food-hoarding hibernators regulate duration of torpor based on the availability of energy. In most fat-storing hibernators such as the large mouse-eared bat (*Myotis myotis*), large body fat stores, i.e., higher body mass, are associated with a reduced number of torpor bouts and higher body temperature during torpor (Wojciechowski et al. 2007). Conversely, lowering body weight by food deprivation prior to hibernation increases torpor bout duration (Wojciechowski et al. 2007). Food-hoarding hibernators depend on the availability and quality of food hoards rather than fat reserve as they feed during arousal phases (French 1988; Williams et al. 2014). Consequently, induction of deep hibernation may depend on reaching a critically low body mass, as found in commercially obtained Syrian hamster (Chayama et al. 2016).

Sex has been found to affect timing of hibernation induction, time spent in torpor and emergence from hibernation as found in Richardson’s ground squirrel (Michener 1978), artic ground squirrel (Buck and Barnes 1999; Buck et al. 2008), European ground squirrel (Millesi et al. 2008), golden-mantled squirrel (Healy et al. 2012) and Anatolian squirrel (Gür and Gür 2015). Females usually enter hibernation later than males to wean their offspring and because juveniles need time to grow before hibernation (Siutz et al. 2016), although the timing of immergence differs among species (Williams et al. 2014). On the other hand, males generally emerge from hibernation earlier in order to maximize their reproductive success. The availability of stored food leads to a decrease in time spent in torpor (Cochet et al. 1999; Fietz et al. 2005). Furthermore, juvenile animals immerge into hibernation later than adult animals, possibly because juveniles need sufficient time to grow and prepare for hibernation (Michener 1984, 1992), although juveniles born late in the season may use torpor prior to hibernation to promote fat accumulation (Giroud et al. 2014).

Regarding diet, especially, the type and concentration of fatty acids present has been suggested to affect the characteristics of hibernation (Geiser 1991; Geiser et al. 2013). Polyunsaturated fatty acids (PUFA), linoleic acid (LA, C18:2; n-6) in particular, lengthen torpor bout duration and lower metabolic rate and body temperature in yellow-bellied marmots (*Marmota flaviventris)*, Alpine marmouts, Eastern chipmunks (*Tamias striatus*), golden-mantled ground squirrels (*Spermophilus lateralis*) among other species (Florant et al. 1993; Thorp et al. 1994; Frank 1992; Bruns et al. 2000), by preserving membrane fluidity through reduction of its melting point (Geiser and Kenagy 1987; Geiser 1991; Hulbert et al. 2005). Fat-storing hibernators including golden-mantled ground squirrels (*Spermophilus lateralis*) and yellow-bellied marmots (*Marmota flaviventris*) have longer and deeper torpor bouts (Geiser and Kenagy 1987; Frank 1992; Florant et al. 1993). In contrast, Eastern chipmunk (*Tamias striatus)*, a food-hoarding hibernator, shortens torpor bouts and extends arousals while exhibiting a higher body temperature when supplied a diet rich in PUFA (Munro et al. 2005). While data on dietary effects of PUFA on torpor duration in Syrian hamster are absent, increased sarcoplasmic linoleic acid (LA) content is associated with augmented activity of the sarcoplasmic reticulum Ca^2+^-ATPase 2a, possibly allowing for torpor at a lower body temperature (Giroud et al. 2013). If so, hamsters fed a PUFA-rich diet would be expected to prolong torpor bout duration, given its dependence on body temperature.

Despite its wide availability, hibernation in commercially obtained Syrian hamster under laboratory conditions is poorly documented. Moreover, whether aforementioned factors affect their quality of hibernation under laboratory conditions is undocumented. This study examined the induction and quality of hibernation in commercially obtained Syrian hamster and identified whether this was dependent on sex and body weight or influenced by a diet enriched in PUFA. To this end, 30 males and 30 female hamsters received either a standard diet or a diet enriched threefold in linoleic acid and parameters including body mass, timing of first torpor bout, number of torpor bouts within the experimental period and time spent in torpor and arousal were analyzed.

## Methods

Animal experiments were approved by the Institutional Animal Ethics Committee of the University of Groningen.

### Experimental procedures and hibernation model

**Fig. 1:**
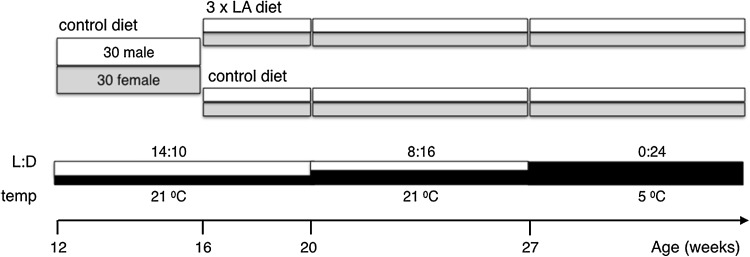
Schematic overview of timing of the experiment. On the *X-axis* the time of switching from one condition to the other is indicated

Thirty male and 30 female Syrian golden hamsters (*Mesocricetus auratus*) were obtained from Harlan (IN, USA, now: Envigo) at the age of 12 weeks at an average body weight of 131 grams (Fig. 1). Information on litter size and littermates was not supplied. Hamsters were individually housed in open top Plexiglas cages from 16 weeks of age and subjected to a hibernation protocol as previously described (Talaei et al. 2011). Hamsters were initially housed at an ambient temperature of 21 °C and a light–dark cycle of 14:10 for 8 weeks. Subsequently, hamsters were subjected to autumn conditions (L:D 8:16, 21 °C) for 7 weeks. When the animals reached an age of 27 weeks, ambient temperature was lowered to 5 °C, and light conditions were changed to continuous dim red light <1 lux. These conditions were maintained for another 9 weeks during which animals received 80 g of food per 14 days. Movement of all animals was continuously monitored with passive infrared detectors (35, Optex, Torrance, CA) fixed on the top of the cages. Data were collected in 2-min bins by a PC-based event-recording system. Previous research from our laboratory has demonstrated that passive infrared movement detection reliably detects hibernation patterns in Syrian hamsters, albeit with overestimation of torpor bout duration of 1.6 h (Oklejewicz et al. 2001). Consequently, periods of complete inactivity lasting >24 h were considered torpor phases. In accord, the shortest two torpor periods lasted 25.3 and 33.5 h, respectively, while all shorter periods of inactivity were <8 h in duration (data not shown). Arousal occurred naturally without outside stimuli or changes in ambient temperature. Start and end of torpor bouts were defined as the timing of the last and first bin showing activity, respectively. Recordings were analyzed until the hamsters reached an aged of 36 weeks, after which they were enrolled in protocols including administration of drugs and/or sampling of tissues. Premature death was defined as death before the end of the hibernation experiment at 36 weeks of age.

### Torpor parameters

The hibernation pattern of each hamster was accessed to quantify the time to first torpor bout, number of torpor bouts and duration of torpor bouts and interbout arousals. Periods of inactivity of >24 h were considered to be torpor phases, any shorter interval of inactivity was taken as a test bout, or as rest or sleeping period.

### Diet

To investigate the effect of linoleic acid on hibernation parameters, female and male Syrian hamsters were equally subdivided at an age of 16 weeks to receive either control diet or a diet three times enriched in linoleic acid, i.e., after being fed the control diet for the first 30 days after arrival. The detailed composition of both diets is provided in Table S1. In brief, control diet consisted of 91% standard diet based on molasses with 46% sugar and supplemented 7% cacao butter. The diet enriched in linoleic acid (3 × LA) contained 7% sunflower oil instead of cacao butter.

### Body mass

Nine body mass measurements of hamsters were recorded about weekly from 16 weeks of age until just prior to switching to winter conditions at 27 weeks of age.

### Statistical analysis

Two-way ANOVA was used to assess the effect of sex and diet on body mass at 18 and 27 weeks of age. RM ANOVA was used to assess differences in body mass, duration of torpor bouts and arousal duration between groups in time. Survival was analyzed using Kaplan–Meier plots; differences between groups were assessed by log-rank test (Mantel–Cox). All other parameters were compared using *t* test, ANOVA or Chi-square tests. All analyses were performed by GraphPad Prism 5.04.

## Results

### The effect of sex and diet on body mass of hamsters

**Fig. 2:**
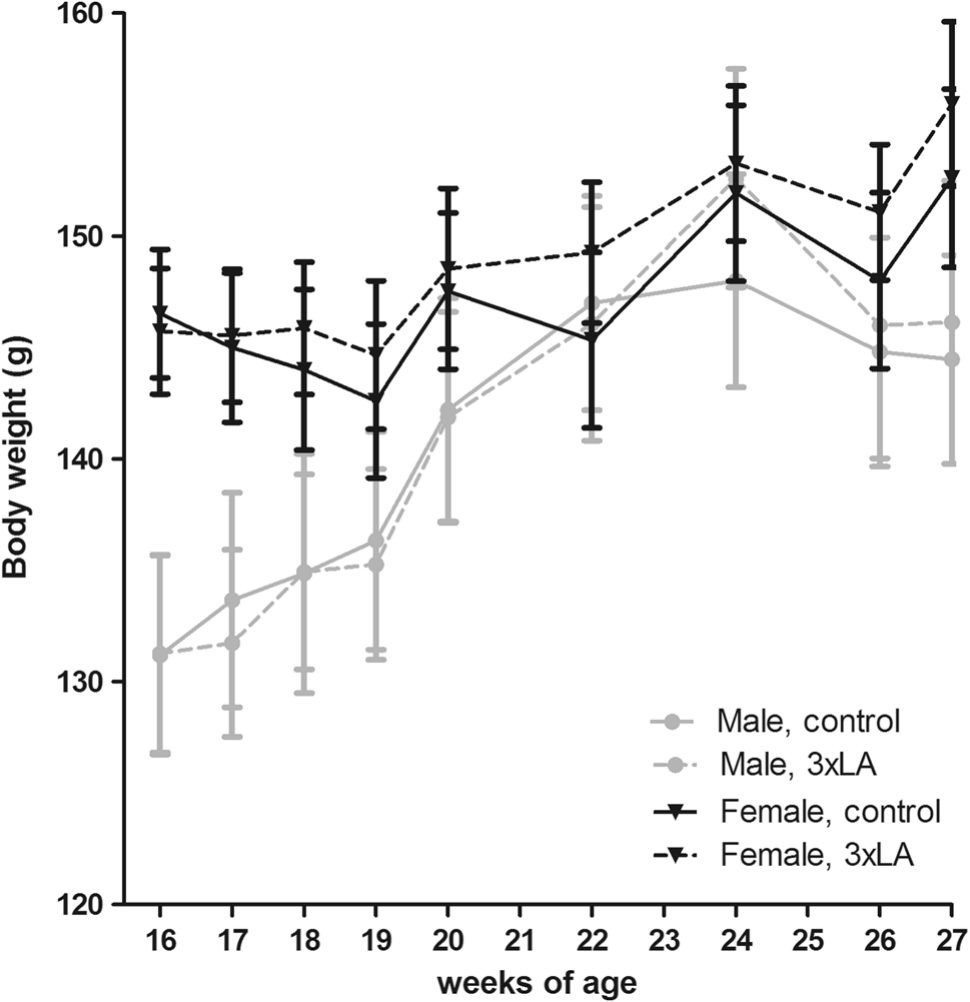
Body mass of hamsters randomized for diet throughout the preparation for hibernation, consisting of subsequent housing at 21 °C and LD 8:16 for 2 weeks, at 21 °C, LD: 8:16 for 9 weeks. Females had significantly higher body mass at 16 weeks than males, but not at 27 weeks of age. *Error bars* represent SEM

Body mass of hamsters was measured about weekly between the age of 16 and 27 weeks, i.e., at the moment of randomization toward control or 3 × LA diet and just prior to switching to winter conditions (Fig. 2). Body mass at the age of 16 and 17 weeks was significantly higher in females than in males (two-way RM ANOVA), with females initially weighing 146.1 ± 2.0 g and males 134.5 ± 2.8 g (two-way ANOVA on sex and diet, *p* = 0.0002). Body weight gradually increased during subsequent ‘autumn’ conditions (L:D = 8:16, 21 °C, *p* < 0.001), particularly in males, resulting in a borderline difference between sexes at week 27 (Females: 155.1 ± 3.2 g, Males: 146.7 ± 3.2 g; two-way ANOVA on sex and diet, *p* = 0.06). Diet did not influence total body mass nor the rate of weight gain in both sexes.

### Premature deaths and the influence of sex and diet

**Fig. 3:**
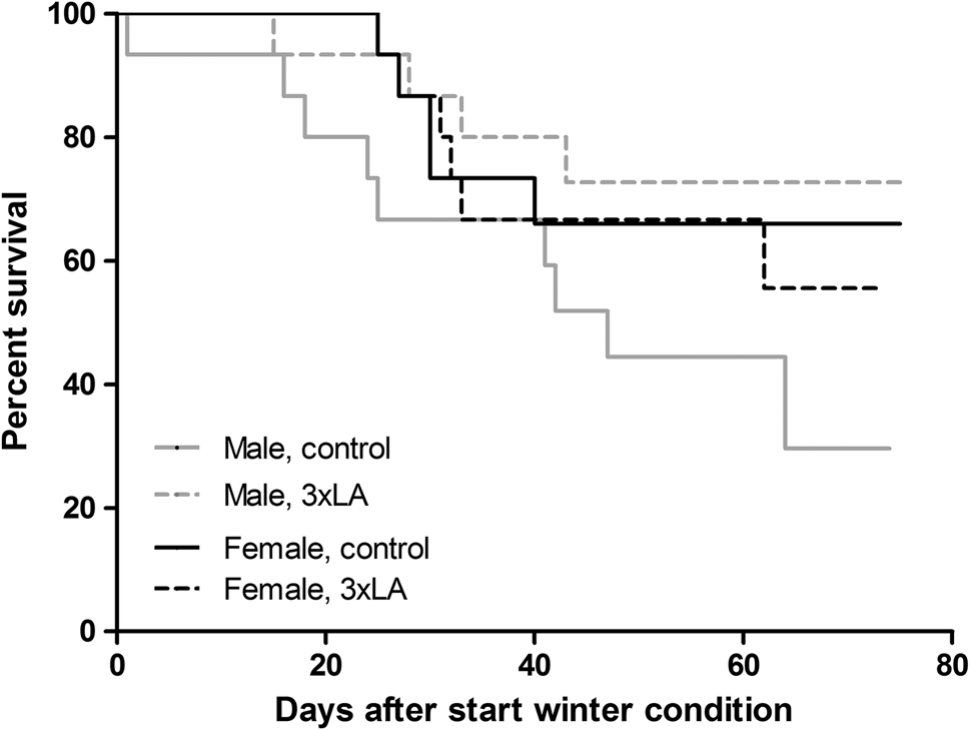
Survival of hamsters in the four experimental groups with time after the onset of winter conditions consisting of 5 °C ambient temperature, LD: 0:24, < 1 lux

**Fig. 4:**
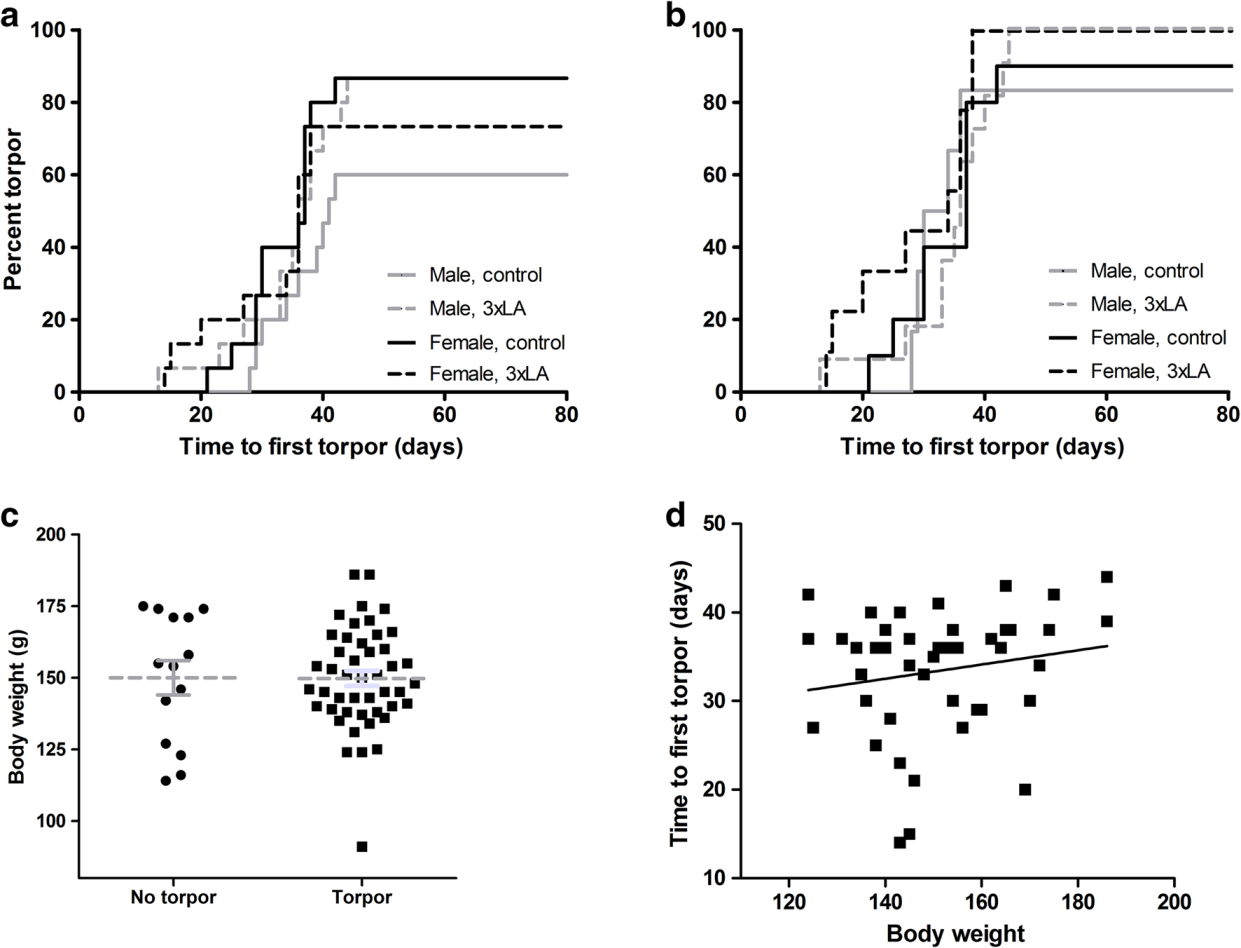
Characteristics of initiation of torpor. **a** The number of days before initiation of the first torpor bout in all hamsters (*n* = 60) expressed as the percentage of all animals, **b** the number of days before initiation of the first torpor bout in the subgroup (*n* = 36) of hamsters that survived hibernation, **c** body weight just prior to switching to ‘winter’ conditions (L:D = 0:24, 5 °C) in animals with and without occurrence of torpor, **d** absence of correlation between the time to first torpor and body weight just prior to switching to ‘winter’ conditions (L:D = 0:24, 5 ^°^C)

We experienced a significant loss of animals after switching to winter conditions (5 °C ambient temperature, LD: 0:24, < 1 lux) before the end of the experiment. In total, 24 out of 60 hamsters (40.0%) died prematurely, nearly all between 2 and 6 weeks after switching to winter conditions (Fig. 3). Male hamsters fed a control diet showed the highest premature deaths (9 out of 15; 60%), although the difference to the other groups did not reach statistical significance (*p* = 0.07). Out of the hamsters that died prematurely, 12 animals (50.0%) never experienced a torpor bout before found dead, which was significantly more than in surviving hamsters from which only two animals did not initiate torpor (5.6%) (Table 1; Fig. 4b, p < 0.005, Chi square test). Body weight just prior to switching to winter conditions did not differ between prematurely deceased and surviving hamsters (Fig. 4c). Likewise, sex and diet did not influence premature death (Table 1). Further, premature death in hamsters that had initiated torpor was observed mostly in animals after torpor bout 2–4 (Table 1). Remarkably, time to first torpor bout and torpor bout duration of prematurely deceased animals did not differ from those in animals that survived (Table 2). Likewise, time to death in animals that refrained from entering torpor did not differ from time to first torpor in animals that subsequently died prematurely and those that survived (Table 2, *p* = 0.2). Deceased animals were invariably found with food and water still available and in the torpid position, i.e., in their nest of hay and fully curled up. Postmortem examination of the animals did not reveal any significant findings.

**Table 1:** Premature death related to number of torpor bouts, diet and sex across experimental groups

# Torpor bouts	# Premature deaths	Females		Males		% Of total deaths
		Control	3 × LA	Control	3 × LA	
0	12	1	4	5	2	50.0
1	3	2	–	1	–	12.5
2	2	1	–	1	–	8.3
3	5	1	1	1	2	20.8
4	0	–	–	–	–	–
5 and more	2	–	1	1	–	8.3
Total	24	5	6	9	4	100

#denotes “number of”; #torpor bouts denotes the number of torpor bouts prior to premature death

**Table 2:** Time to first torpor bout and torpor bout duration of premature deaths versus survivors for first three torpor bouts

Torpor bout number	Time to first bout (days)		Duration (h)	
	Premature deaths	Survivors	Premature deaths	Survivors
0	31.7 ± 3.1* (*n* = 12)	–	–	–
1	36.6 ± 1.6* (*n* = 12)	32.0 ± 1.4 (*n* = 34)	52.8 ± 2.24 (*n* = 3)	52.5 ± 2.0 (*n* = 34)
2	–	–	60.7 ± 5.6 (*n* = 2)	64.9 ± 3.4 (*n* = 34)
3 and more	–	–	74.3 ± 5.5 (*n* = 7)	72.8 ± 1.2 (*n* = 34)

*Denotes time to death

### The effect of sex and diet on torpor induction

The time to first torpor bout after onset of winter conditions was accessed in all hibernating hamsters (Fig. 4a) and in hamsters that survived the whole experimental period (Fig. 4b). In both groups, neither sex nor diet influenced the timing of the first torpor bout. Further, body mass just prior upon start of winter conditions and diet did not differ between animals that did and did not initiate torpor (Fig. 4c). Finally, body mass did not relate with the timing the first torpor bout in hibernating animals (Fig. 4d, *R* ^2^ = 0.03, *p* = 0.25).

### The effect of sex and diet on torpor and arousal duration

**Fig. 5:**
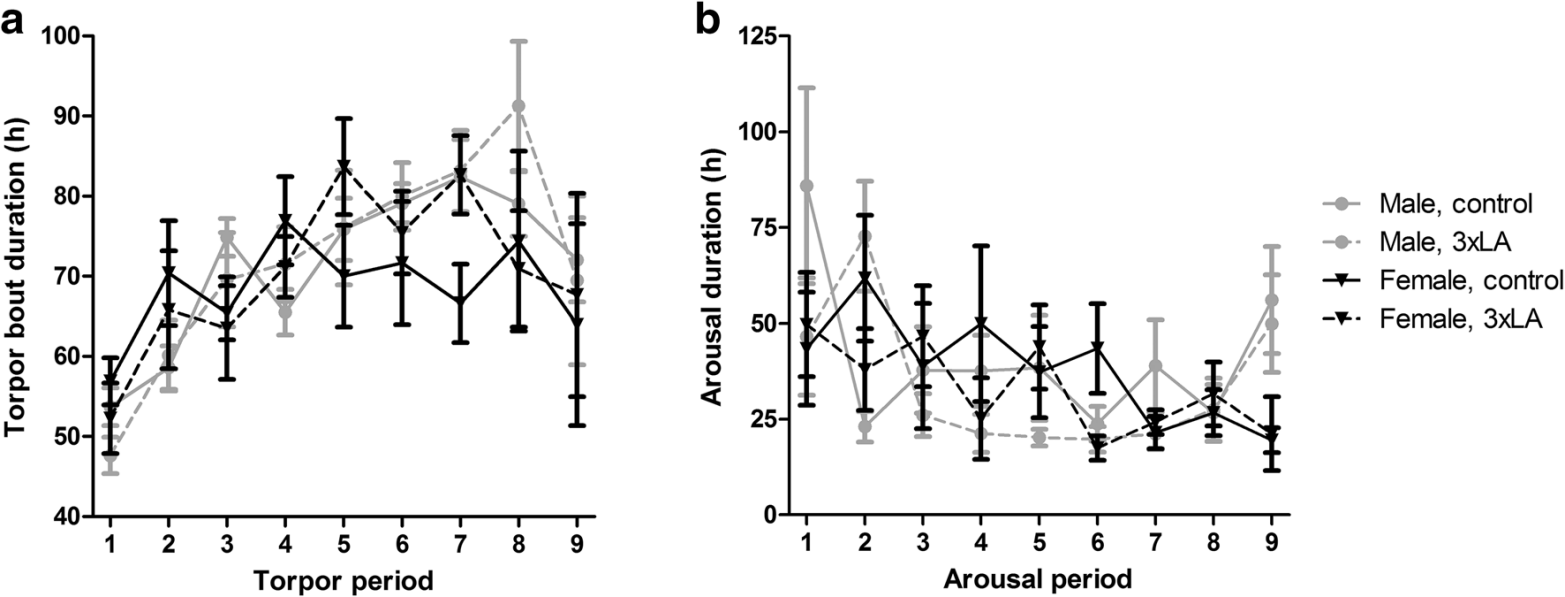
Duration of torpor bouts and arousals. Torpor bout duration showed a gradual increase during the first bouts, whereas arousal duration declined. No differences were observed between the four experimental groups for torpor bout duration (**a**), or for duration of arousal periods (**b**)

The effect of sex and diet on the duration of torpor and arousal bouts was analyzed (Fig. 5). The duration of each successive torpor bout increased until the 5th torpor bout. Thereafter the number of hours per torpor bout remained constant averaging about 80 h. Neither sex nor diet influenced the duration of torpor bouts. An inverse pattern was found for arousal, as its duration decreased gradually until reaching a plateau from the third arousal onwards. Similarly to torpor bout duration, sex and died did not affect the duration of arousal periods.

## Discussion

Despite the use of Syrian hamster in hibernation research (Giroud et al. 2013; Weitten et al. 2013; Horwitz et al. 2013; Antón-Fernández et al. 2015; León-Espinosaet al. 2016; Chayama et al. 2016), the influence of fundamental parameters such as body mass, sex and diet on the characteristics of hibernation is poorly documented in this species. Moreover, although in various hibernating species the effect of body mass, sex and diet on survival on the timing of hibernation and duration of torpor and arousal has been well documented in the wild, their effect is scantly documented under laboratory conditions (see Introduction for references). Here we documented effects of these parameters on the quality of hibernation in commercially obtained Syrian hamster. Initial body mass at age 16 weeks was smaller in males, who gradually caught up with females during the next 11 weeks of ad libitum feeding, irrespective of diet. Our principle finding is that hamsters experienced a high rate of premature death, both in animals that did and did not initiate torpor, which was unrelated to body weight, sex and diet. Furthermore, timing of induction of hibernation and duration of torpor and arousal was not affected by body weight, sex or diet. Thus, commercially obtained Syrian hamsters subjected to winter conditions showed poor survival, while body weight, sex and diet did not affect hibernation parameters.

### Premature death and factors influencing it

This is the first study reporting a very high (40%) premature death rate in commercially obtained Syrian hamster hibernating under laboratory conditions. Notably, premature death occurred for 50% in animals that had not entered into torpidity prior to being found dead, which signifies that they either died during their first torpor bout or, alternatively, while maintaining euthermy under winter conditions. Similarly, discrimination between premature death during torpor or euthermy is also impossible in animals that did successfully endured torpid period(s). However, these animals, completing two or more torpor bouts, displayed regular torpor patterns until the time of their demise, suggesting that they died while in torpor or during early arousal. Strikingly, time to death in animals that did not enter torpor was similar to time to first torpor bout in prematurely deceased animals that did enter torpor, both being similar to time to first torpor in hibernating hamsters. This observation, together with their posture and absence of abnormalities during postmortem examination, may signify that prematurely dying hamster all entered torpor, but lacked the signal or ability to arouse during their first or one of the subsequent torpor bouts. Body mass, sex and diet did not influence survival. It is hard to comment on the reason for this excessive premature death rate, as to our knowledge there are no published data in commercially obtained Syrian hamsters subjected to winter conditions. However, the high mortality rate in our hamsters is not entirely unprecedented in hamsters from local breeding colonies. Terada and Ibuka (2000) reported a mortality of 60% in a 5 months’ period in hamsters entering hibernation at an age of 14 months, while adult and younger animals (still) showed 20% mortality, while Oklejewicz et al. (2001) observed 25% mortality in tau mutant hamsters. As these and our reports represent three different sources of animals in three different laboratories, it suggests that death during torpor may be an intrinsic property of Syrian hamster—perhaps related to their inbred nature. Commercial production of hamsters often involves prolonged breeding at high reproduction rate amounting 4–6 litters per female of 11 offspring on average (Hoosier and McPherson 1987). Their highly similar genetic background may thus have produced selection against the capacity to successfully hibernate, as this trait is likely unwanted by commercial breeders. Such explanation could be confirmed by a genetic screen on premature deaths versus survivors, as numerous other factors may have determined premature death as well. Indeed, an early report suggests that hibernation traits in Syrian hamsters are governed by a few number of genetic loci, as selective breeding of hibernating and non-hibernating Syrian hamster results already in the F3 generations in lines with a marked difference in propensity to hibernation (Chaffee 1966). Nevertheless, we cannot exclude involvement of additional factors, such as a viral infection or other unusual environmental perturbations affecting hibernating hamsters. Studying the influence of environmental factors will require comparison of SPF hamsters subjected to hibernation induction with littermates kept under long day length.

### Quality of hibernation and factors influencing it

None of the factors previously documented to influence the quality of hibernation was found to affect time to initiation of torpor or duration of torpor bout and arousal. Even though factors such as body mass, sex and diet composition play a role in the wild (see Introduction for references), their effect is poorly documented under laboratory conditions, hampering interpretation of our data. As Syrian hamsters are food-hoarding hibernators, they only moderately fatten up in preparation of hibernation (Bartness and Wade 1985), and rather feed during periodic arousals (French 2000; Vander Wall 1990). Therefore, availability and quality of food (approximate composition and fatty acid composition) primarily affect torpor characteristics. The ad libitum availability of high-quality food pellets also enabled male hamsters to catch up in body mass with females prior to the induction of hibernation.

In the wild, female European hamster were found to enter hibernation later than males, likely because of weaning their offspring and because young need time to grow before the onset of hibernation (Siutz et al. 2016). Females also have larger food stores than males, who spent less time hoarding to increase the chance of reproduction and because of a frequent change of burrows (Siutz et al. 2016). Because of excess supplies, female hamsters spend less time in torpor than males. Further, males emerge from hibernation earlier to complete spermatogenesis and defend territories (Zervanos et al. 2014). None of these traits were found in our laboratory animals. Possibly they are suppressed by the individual housing or result from commercial breeding.

The effects of PUFA on hibernation are well documented (Munro and Thomas 2003; Ruf and Arnold 2007). More specifically, increased PUFA content in the diet of wild captured animals showed a positive effect on torpor bout duration, minimum body temperatures tolerated and energy savings (Frank 1992; Geiser et al. 1994; Thorp et al. 1994). In Syrian hamster, higher proportion of linoleic acid in the sarcoplasmic reticulum increases the activity of SERCA in torpor and thus determines the minimum body temperature tolerated (Giroud et al. 2013). Based on these findings, it was expected that hamsters fed a linoleic acid enriched diet would also exhibit longer torpor bouts, because of lower body temperature in torpor. However, this was not confirmed by our study. The prolongation of torpor bout duration in linoleic acid supplied hibernating animals may be species specific. However, in face of the prevailing assumption that it is caused by maintaining membrane fluidity, this explanation appears unlikely. In addition, higher amount of PUFA in the diet may not always be necessary to maintain lipid fluidity. Specific hibernators with limited access to PUFA-rich diets, including insectivores, may have developed alternative mechanisms to maintain lipid fluidity (Schalk and Brigham 1995). In addition, PUFA also promote hydrogen peroxidase formation and deplete antioxidants, vitamin C and cholesterol, thus balancing the effect on membrane fluidity depending on their intake (Munro and Thomas 2003). Furthermore, species with ready access to PUFA-rich diets seek a specific target level that limits oxidative stress. Therefore, when animals are supplied with PUFA enriched diets that result in PUFA levels exceeding those found in the wild, its beneficial effects on torpor characteristics may be lost (Munro and Thomas 2003).

### Body weight differences between males and females

Males displayed significantly lower body weights at 16 weeks of age than females, but caught up with females during the subsequent 9 weeks of ad libitum feeding irrespective of diet, while females only showed a modest increase in weight. A probable explanation for the weight difference between sexes until the age of 16 weeks is the housing of the animals. Animals had been shipped in groups of 15 and were subsequently housed in groups of five animals of the same gender. Previously, it was demonstrated that group housing compared to individual housing precipitates a larger gain in body weight in females (Meisel et al. 1990) than in males (Arnold and Estep 1990), amounting 42 and 19%, respectively. However, Gattermann et al. (2002) report a 14% increase in weight in group housing irrespective of sex, but these results may have been influenced by a higher aggression in females forcing separation of groups. From the age of 16 weeks onwards, our hamsters were randomized for diet and individually housed and males quickly gained weight. Thus, our data suggest that the effect of group housing on body weight is preserved after switching to individual housing in females. Further, in males, switching to individual housing may even undo the factor causing their less weight gain during initial group housing.

In summary, our study documents a large proportion of premature death in commercially bred Syrian hamster of both sexes following induction of hibernation, irrespective of body weight or diet composition. Notably, 50% of the hamsters that died had not hibernated, thus representing premature death following the exposure to cold ambient temperatures, whereas the remainder demised after completing 1–6 torpor bouts. Further, while documented in wild or wild captured hoarding and fat-storing hibernators, body weight, sex and diet composition did not influence the quality of hibernation in our Syrian hamsters. Although the reason(s) remain elusive, possibly the long-term commercial breeding from a confined genetic background has selected against the hibernation phenotype.

## Electronic supplementary material

Below is the link to the electronic supplementary material.

Supplementary material 1 (DOCX 100 KB)

## Abbreviations

3xLA: Diet enriched with threefold linoleic acid
PUFA: Polyunsaturated fatty acids
