# Supplementary material for: The influence of sex and diet on the characteristics of hibernation in Syrian hamsters
Source: J Comp Physiol B. 2017 Mar 21;187(5):725–34. doi: 10.1007/s00360-017-1072-y (PMC5486544; doi:10.1007/s00360-017-1072-y)
Supplement: Supplementary file 1 — Supplementary material 1 (DOCX 100 KB) [file 360_2017_1072_MOESM1_ESM.docx]

Table S1. Composition of control and 3xLA diets.

|  | Control diet  (Cacao butter 7%) | 3xLA enriched  (Sunflower oil 7%) |
| --- | --- | --- |
| Cr.Prot g/kg | 234.8 | 234.8 |
| Cr. Fat g/kg | 128.8 | 128.5 |
| Cr.Fiber g/kg | 29.1 | 29.1 |
| Minerals g/kg | 49.2 | 49.2 |
| Moisture g/kg | 99.2 | 99.5 |
| Sug.+St. g/kg | 383.6 | 383.6 |
| Nfree ex g/kg | 454.0 | 454.0 |
| Dry Mat. g/kg | 900.8 | 900.5 |
| Lysine g/kg | 10.7 | 10.7 |
| Methion. g/kg | 3.6 | 3.6 |
| Cystine g/kg | 3.0 | 3.0 |
| Threonin g/kg | 7.7 | 7.7 |
| Tryptoph g/kg | 2.2 | 2.2 |
| Isoleuc. g/kg | 7.8 | 7.8 |
| Arginine g/kg | 14.3 | 14.3 |
| Phenylal g/kg | 8.7 | 8.7 |
| Histidin g/kg | 4.6 | 4.6 |
| Leucine g/kg | 14.5 | 14.5 |
| Tyrosine g/kg | 5.6 | 5.6 |
| Valine g/kg | 10.6 | 10.6 |
| Alanine g/kg | 15.4 | 15.4 |
| Asp.acid g/kg | 17.1 | 17.1 |
| Glut.ac. g/kg | 37.5 | 37.5 |
| Glycine g/kg | 24.4 | 24.4 |
| Proline g/kg | 19.8 | 19.8 |
| Serine g/kg | 9.5 | 9.5 |
| Calcium g/kg | 8.3 | 8.3 |
| Phos.tot g/kg | 5.0 | 5.0 |
| Phos.dig g/kg | 2.0 | 2.0 |
| Potass. g/kg | 7.8 | 7.8 |
| Magnes. g/kg | 1.2 | 1.2 |
| Sodium g/kg | 2.6 | 2.6 |
| Chlorine g/kg | 3.3 | 3.3 |
| Sulfur g/kg | 1.0 | 1.0 |
| **Gross En kcal** | **4509.0** | **4509.0** |
|  |  |  |
| C8-C12:0 g/kg | 0.1 | 0.1 |
| C14:0 g/kg | 0.5 | 0.4 |
| **C16:0 g/kg** | **13.4** | **26.7** |
| C16:1 g/kg | 0.8 | 0.7 |
| **C18:0 g/kg** | **6.8** | **27.8** |
| **C18:1 g/kg** | **30.7** | **39.1** |
| **C18:2 g/kg** | **63.1** | **20.4** |
| C18:3 g/kg | 7.5 | 7.2 |
| C20-C22 g/kg | 1.1 | 0.7 |
|  |  |  |
| Vitamins /kg: |  |  |
| Vit. A IU | 14600 | 14600 |
| Vit. D3 IU | 1420 | 1420 |
| Vit. E IU | 80.6 | 80.6 |
| Vit. K3 mg. | 2 | 2 |
| Vit. B1 mg. | 14 | 14 |
| Vit. B2 mg. | 9 | 9 |
| Vit. B6 mg. | 13 | 13 |
| Niacin mg. | 37 | 37 |
| Pant.ac. mg. | 20 | 20 |
| Vit.B12 mcg. | 48 | 48 |
| Folic.ac mg. | 4 | 4 |
| Choline mg. | 131 | 131 |
| Biotin mcg. | 518 | 518 |
| Starch g/kg | 342 | 342 |
| Sugars g/kg | 41 | 41 |
| Lactose g/kg | 14 | 14 |
| NDF g/kg | 91 | 91 |
| ADF g/kg | 36.8 | 36.8 |
| Hemicel. g/kg | 55 | 55 |
|  |  |  |
| Trace elements /kg |  |  |
| Iron mg. | 108 | 108 |
| Mangan. mg. | 50 | 50 |
| Zinc mg. | 73.8 | 73.8 |
| Copper mg. | 17.1 | 17.1 |
| Cobalt mg. | 1.6 | 1.6 |
| Iodine mg. | 0.2 | 0.2 |
| Selenium mg. | 0.1 | 0.1 |
